# Superelastic Graphene Aerogel/Poly(3,4-Ethylenedioxythiophene)/MnO_2_ Composite as Compression-Tolerant Electrode for Electrochemical Capacitors

**DOI:** 10.3390/ma10121353

**Published:** 2017-11-24

**Authors:** Peng Lv, Yaru Wang, Chenglong Ji, Jiajiao Yuan

**Affiliations:** College of Electronic and Optical Engineering, Nanjing University of Posts & Telecommunications, Nanjing 210023, China; wangyr@njupt.edu.cn (Y.W.); jicl@njupt.edu.cn (C.J.); Yuanjj@njupt.edu.cn (J.Y.)

**Keywords:** graphene, poly(3,4-ethylenedioxythiophene), MnO_2_, composite, compression-tolerant, electrode, electrochemical capacitors

## Abstract

Ultra-compressible electrodes with high electrochemical performance, reversible compressibility and extreme durability are in high demand in compression-tolerant energy storage devices. Herein, an ultra-compressible ternary composite was synthesized by successively electrodepositing poly(3,4-ethylenedioxythiophene) (PEDOT) and MnO_2_ into the superelastic graphene aerogel (SEGA). In SEGA/PEDOT/MnO_2_ ternary composite, SEGA provides the compressible backbone and conductive network; MnO_2_ is mainly responsible for pseudo reactions; the middle PEDOT not only reduces the interface resistance between MnO_2_ and graphene, but also further reinforces the strength of graphene cellar walls. The synergistic effect of the three components in the ternary composite electrode leads to high electrochemical performances and good compression-tolerant ability. The gravimetric capacitance of the compressible ternary composite electrodes reaches 343 F g^−1^ and can retain 97% even at 95% compressive strain. And a volumetric capacitance of 147.4 F cm^−3^ is achieved, which is much higher than that of other graphene-based compressible electrodes. This value of volumetric capacitance can be preserved by 80% after 3500 charge/discharge cycles under various compression strains, indicating an extreme durability.

## 1. Introduction

Recently, the great development of wearable electronics not only enriches our daily lives, but also requires the matched energy storage devices, which should withstand various high-level strains, such as bending, stretching, and compression [1,2]. Electrochemical capacitors (ECs) outstand as a promising solution due to their high power density, rapid charge rate, and long cycle life [3]. Previous reports suggest that the electrode of ECs is one of the major limiting factors in maintaining the capacity performances under high-level strains [4,5]. In the past decade, there are plentiful studies and achievements for the functional electrodes of ECs with bendable and stretchable functions, paving the way for the application in strain-tolerant energy devices [6,7]. Nevertheless, design and fabrication of compression-tolerant electrodes of ECs rarely appears, although the compressive strain is one of the most important influences for the performances of the electrodes. However, most of the conventional electrodes decrease or lost their functions due to deformation or destruction of electrode material at high compression strain [8]. For the porous and low-density materials, their intrinsic electrical and transport properties are easy to deteriorate when they are deformed at high-level compressive strains. For the highly dense materials, it is difficult to offer continuous ion pathways and facile access to storage sites when they are highly compacted.

Recently, some graphene-based monoliths (aerogels/sponges/foams) with ordered porous structure (such as honeycomb-like cellular structure [9,10], bubble structure [11], and multi-arch structure [12]) show ultra-compressible ability (maximum recoverable compressive strain reaching 90–99%). This excellent mechanical property arises from the integrated graphene cell walls and the ordered porous architecture. In the cell walls, the tightly integrated multilayered structure can maximize the π-π interaction between graphene layers and thus greatly enhance strength and elastic stiffness of cell walls. And the cells organized in ordered architecture provide the maximum elastic modulus and strength for the graphene monoliths [13,14]. Thus, the ultra-compressible graphene-based monoliths show promising potential for the compression-tolerant electrodes for ECs [15,16]. There are already some reports about the application of ultra-compressible graphene monoliths as compression-tolerant electrodes (Table 1). It can be classified into two categories: compressible graphene aerogel electrodes [2,17] and compressible graphene/pseudomaterial composite electrodes [18,19,20]. Although some progresses have been achieved, these compressible electrode materials still cannot possess high capacitance and high compression-tolerant ability simultaneously. For graphene aerogel electrodes, although their maximum compressive strain is relative high (reaching 90%), the specific capacitances of them are still too low due to the electric double-layer storage mechanism of carbon materials [2,17]. For graphene/pseudomaterial composite electrodes, although the pseudomaterials contribute the high pseudocapacitance for the electrodes, their recoverable compressive strains are still too low. For example, the maximum compressive strain of melamine foam/graphene/polypyrrole sponge is only 75% [18]; graphene/polypyrrole foam even cannot retain compressible at dry condition [19]; and graphene-carbon nanotube/MnO_2_ aerogel shows recoverable compressive strain of only 50% [20]. Therefore, for achieving the graphene-based compressible electrodes with both high capacitances and high recoverable compressive strains, it is necessary to design a novel structure.

**Figure 1:**
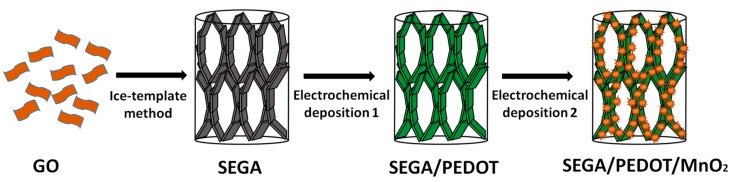
Schematic diagram of preparation compressible SEGA/PEDOT/MnO_2_ composite.

Herein, we report a ternary composite electrode with both high capacitances and high compression-tolerant ability by electrodepositing poly (3,4-ethylenedioxythiophene) (PEDOT) and MnO_2_ into the superelastic graphene aerogel (SEGA) (Figure 1). SEGA as the conductive backbone contributes its high compressibility to the electrode. PEDOT as the interface layer between graphene and MnO_2_ can reduce their interface resistance. And the cell walls of SEGA can be reinforced by the PEDOT coating layer, leading to the further improvement of compressibility. MnO_2_ deposited on the cell walls of SEGA/PEDOT produces high pseudocapacitance for the composite electrode. We tested the recoverable compressive strains and the durability of SEGA/PEDOT/MnO_2_ composite by the compression/release measurements, and characterized its electrochemical performances using the three-electrode system. In addition, we also fabricated the all-solid-state ECs based-on SEGA/PEDOT/MnO_2_ electrodes to demonstrate the change of the gravimetric capacitance and volumetric capacitance of the electrodes at various compressive strains.

## 2. Experimental Section

### 2.1. Preparation of Ultra-Compressible SEGA/PEDOT/MnO_2_ Composite

Graphene oxide (GO) was prepared according to the modified Hummers’ method. The SEGA was firstly prepared by the ice template method [9]. In a typical synthesis procedure, GO aqueous dispersion (4 mg mL^−1^, 12 mL) with 100 mg l-ascorbic acid was poured into a flat-bottomed glass test tube and heated for 20 min at 95 °C to obtain the partially reduced graphene hydrogel. Then the hydrogel was treated by the freeze-thaw process in the refrigerator (−23 °C) and room temperature. Subsequently, further reduction process for the freeze-recast hydrogel was performed for 6 h at 95 °C by the initial l-ascorbic acid. Finally, the completely reduced hydrogel was dried at 60 °C for 24 h to obtain the SEGA.

The electrochemical deposition of PEDOT into SEGA was performed by the galvanostatic method using a three-electrode system, where the SEGA was used as the working electrode, a platinum electrode as the counter electrode, and a Ag/AgCl electrode as the reference electrode. Aqueous solution consisting of 0.01 M 3,4-ethylenedioxythiophene and 0.005 M sodium dodecyl sulfate (surfactant) was used as the precursor solution. And the pH value of precursor solution was adjusted by slowly adding *ρ*-toluenesulfonic acid (dopant) to pH = 1. The deposition was performed at a current of 1 mA cm^−2^ for 2 min. After the working electrode was washed with distilled water and dried at 60 °C for 8 h, the SEGA/PEDOT binary composite was obtained.

MnO_2_ was subsequently deposited into SEGA/PEDOT composite. The aqueous deposition bath containing 0.01 M KMnO_4_ and 0.1 M LiClO_4_ was dispersed adequately under ultrasonication prior to use. Electrochemical deposition was carried out using a three-electrode system, in which the SEGA/PEDOT acted as the working electrode, a Pt foil served as the counter electrode, and a saturated calomel electrode (SCE) as the reference electrode. The deposition process was performed by applying a constant reduction potential of −1.2 V vs. SCE for 20 min. After washing the working electrode with distilled water and drying at 60 °C for 12 h, the SEGA/PEDOT/MnO_2_ ternary composite was obtained. For comparison, SEGA/MnO_2_ composite was also prepared by the same deposition conditions.

The mass contents/mass loading of PEDOT and MnO_2_ in the ternary composite calculated from the weight changes of samples before and after each deposition steps were 18.8 wt %/2.1 mg cm^−2^ (PEDOT) and 58.6 wt %/6.5 mg cm^−2^ (MnO_2_), respectively (Table S1).

### 2.2. Characterizations

The chemical structure of the samples was investigated by Fourier transform infrared spectroscopy (FTIR, Nicolet 520, Thermo Scientific, Waltham, MA, USA), and X-ray photoelectron spectroscopy (XPS, PHI 1600 spectroscopy, PerkinElmer, Waltham, MA, USA). The crystal structure of the composites was characterized by X-ray diffraction (XRD, Philip X’ Pert Pro MPP, PANalytical B.V., Almelo, The Netherlands) using a Cu-Kα radiation (λ = 1.5418 Å). The microstructure of the samples was observed by the scanning electron spectroscopy (SEM, S-4800, Hatchi, Tokyo, Japan) equipped with energy dispersive spectroscopy (EDS, Hatchi, Tokyo, Japan). Compression/release measurements were performed on a single-column static instrument (model 5943, Instron, Norwood, MA, USA) using a 10 N load cell and strain control mode with a strain rate of 5% per second. During the measurements, the testing samples were placed between the two flat-surface compression stages of the instrument. The dimension of compression testing samples was 5 mm × 8 mm × 8 mm.

Electrochemical characterizations including cyclic voltammetry (CV), galvanostatic charge-discharge (GCD) and electrochemical impedance spectroscopy (EIS) were performed using the CHI660E electrochemical workstation (CH Instruments Inc., Austin, TX, USA). The electrochemical measurements of individual electrode were performed in the three-electrode system with 1 M H_2_SO_4_ aqueous electrolyte. The SEGA/PEDOT/MnO_2_ composite, Pt wire and Ag/AgCl (purchased from CH Instruments Inc., Austin, TX, USA) were used as working electrode, counter electrode and reference electrode, respectively. The specific capacitance (*C_s_*) was calculated from the GCD curves according to the following equation:

*C_s_* = *I*×∆*t*/*m*×∆*V* (1)

where *I* is the constant discharge current, ∆*t* is the discharging time, *m* is the total mass of the working electrode (including the SEGA, PEDOT and MnO_2_), ∆*V* is the voltage drop upon discharging.

To investigate the electrochemical performances of the SEGA/PEDOT/MnO_2_ electrodes under compressive conditions, we assembled the compressible all-solid-state ECs [21,22,23]. Firstly, the PVA/H_2_SO_4_ gel electrolyte was prepared by mixing PVA powder, H_2_SO_4_, and deionized water according to the mass ratio of 5:4:50. Then the mixture was stirred for 40 min at 80 °C to form a clear electrolyte. Secondly, SEGA/PEDOT/MnO_2_ composites with a thickness of ~5 mm were immersed into the PVA/H_2_SO_4_ gel electrolyte for 10 min. After evaporating of excess water, the electrolyte adhered on the surface of cell walls of the ternary composite. Then two pieces of the composites two electrodes with similar weight were placed onto two poly(ethylene terephthalate) substrates with Au (~100 nm), respectively. One piece of porous separator (model 3501, Celgard, Charlotte, NC, USA) was also infiltrated with PVA/H_2_SO_4_ gel electrolyte. The compressible all-solid-state ECs were obtained by assembling the as-prepared two electrodes with identical or very close weight sandwiched with the separator under pressure (~1 N). Finally, the device was kept at 45 °C for 24 h to remove excess water in the electrolyte.

The gravimetric capacitance (*C_g_*) and volumetric capacitance (*C_vol_*) of the SEGA/PEDOT/MnO_2_ electrodes in the compressible ECs were calculated from the GCD curves using the following Equations (2) and (3):

*C_g_* = 4 × *I* × ∆*t*/*m* × ∆*V* (2)

*C_vol_* = *ρ* × *C_g_* (3)

where *I* is the constant discharge current, ∆*t* is the discharging time, *m* is the total mass of two electrodes, ∆*V* is the voltage drop upon discharging, *ρ* is the density of the SEGA/PEDOT/MnO_2_ composite under various compressive strains.

## 3. Results and Discussion

**Figure 2:**
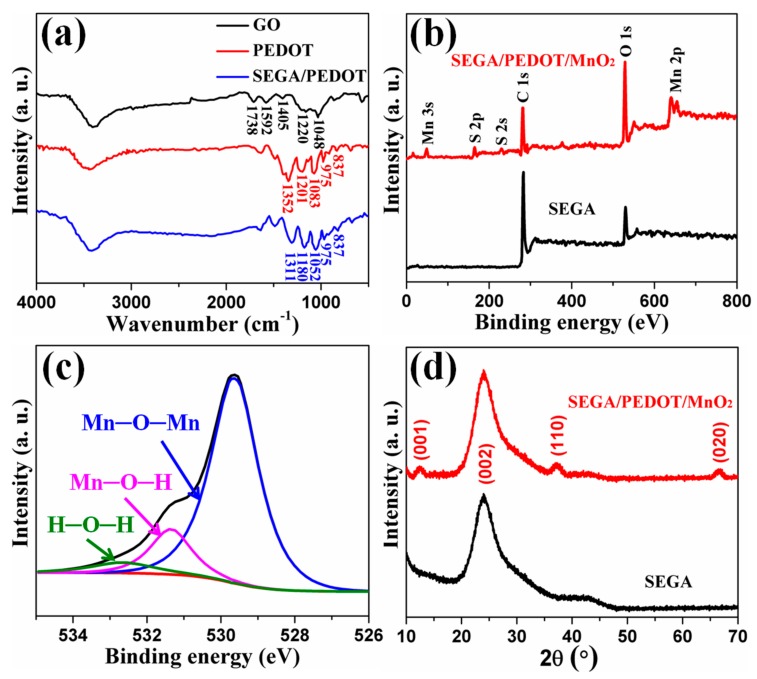
(**a**) FTIR spectrum of GO, PEDOT and SEGA/PEDOT composite; (**b**) XPS spectrum of SEGA and SEGA/PEDOT/MnO_2_ composite; (**c**) O 1s spectra of SEGA/PEDOT/MnO_2_ composite; (**d**) XRD patterns of SEGA and SEGA/PEDOT/MnO_2_ composite.

The composition of the SEGA/PEDOT composite was firstly studied by FTIR (Figure 2a). For PEDOT, the peak at 1352 cm^−1^ originates from C–C and C–C stretching vibrations of the quinoid structure in the thiophene ring, peaks at 1201 and 1083 cm^−1^ are attributed to C–O–C bond stretching, and peaks at 975 and 837 cm^−1^ are corresponding to the C–S bond in the thiophene ring [24,25]. And the spectrum of SEGA/PEDOT consists of similar functional groups present in PEDOT. But the peak corresponding to C=C and C–C stretching vibrations in thiophene ring is red-shifted to 1311 cm^−1^, and the peaks corresponding to C–O–C bond stretching are red-shifted to 1180 and 1052 cm^−1^, which is attributed to the π-π interactions between graphene and PEDOT [26]. In addition, the peak at 1738 cm^−1^ corresponding to –C=O/–COOH stretching peak of GO disappears, indicating that the reduction process removes most of the carbonyl groups. XPS was performed to characterize the chemical structure of SEGA/PEDOT/MnO_2_ composite. Compared with the XPS spectra of SEGA, the full spectra of SEGA/PEDOT/MnO_2_ composite reveals additional S 2p, S 2s, Mn 2p and Mn 3s peaks (Figure 2b), indicating the presence of PEDOT and MnO_2_. As shown in Figure S1, the Mn 2p region consists of a spin–orbit doublet of Mn 2p_3/2_ with a binding energy of 653.5 eV and Mn 2p_1/2_ with a binding energy of 642.1 eV, which are characteristic peaks of a mixed-valence manganese system (Mn^4+^ and Mn^3+^) [6,20]. The high-resolution XPS O 1s spectra (Figure 2c) is deconvoluted into three constituents at the binding energy of 529.9 eV (Mn–O–Mn bond of the tetravalent oxide), 531.3 eV (Mn–O–H bond of the hydrated trivalent oxide), and 532.3 eV (H–O–H bond of the residual water) [27,28]. The area ratios are 0.70, 0.21, and 0.09 for Mn–O–Mn, Mn–O–H, and H–O–H, respectively. Thus, the dominated oxidation state of manganese oxide in composite is tetravalent. XRD was performed to study the crystal phase of MnO_2_ in the composites. As shown in Figure 2d, the typical diffraction peaks at 12.4°, 37.5°, 66.8° can be indexed to the crystal planes of (001), (110), and (020) in birnessite-type MnO_2_ (JCPDS card No. 42-1317) [29,30,31], respectively. These peaks are broad and weak, suggesting that the crystallinity of electrodeposited MnO_2_ in the composite is relatively poor. The peak at 12.4° indicates the presence of a typical layer crystal structure. In addition, the broad diffraction peak at ca. 24.2° can be indexed to the (002) reflection of graphene.

**Figure 3:**
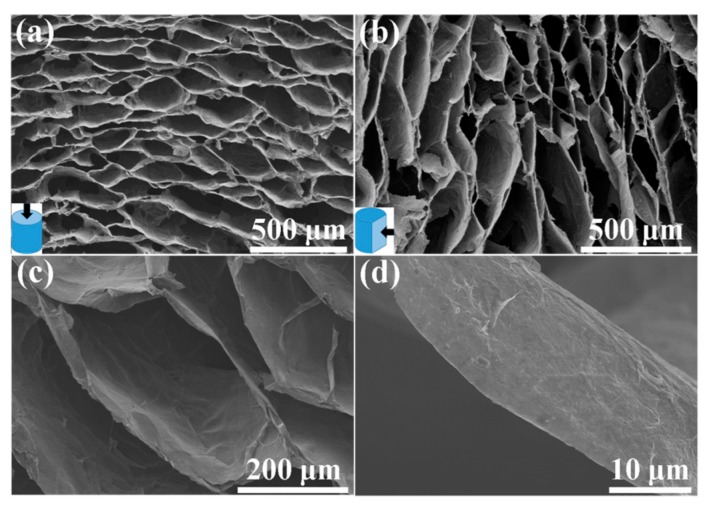
SEM images of (**a**) cross-section and (**b**) vertical-section of SEGA; (**c**,**d**) SEM images at high magnifications of SEGA.

Figure 3 shows the SEM images of as-prepared SEGA. It can be seen that SEGA possesses the macro-porous, honeycomb-like and oriented cellular structure at both cross-section view and vertical-section view (Figure 3a,b). The graphene sheets are tightly packed and well oriented in parallel manner to build the cellar walls of SEGA (Figure 3c,d). As reported in the previous literatures [9,32,33,34], the honeycomb-like and oriented cellar structure enhances the mechanical strength of the graphene cells and bring the graphene aerogel ultra-compressible ability. In addition, the cell dimension in SEGA is as large as hundreds of micrometers, which is in favor of the impregnation of precursor solution during the subsequent deposition processes.

**Figure 4:**
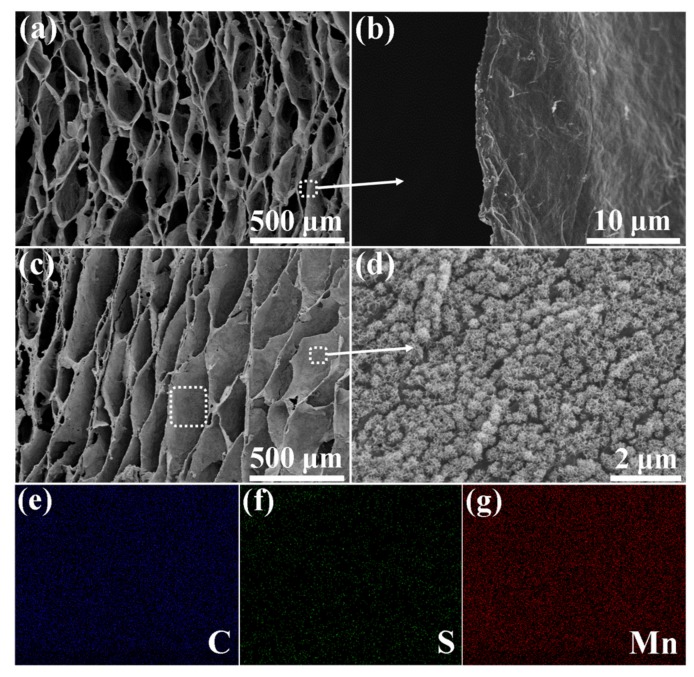
SEM images of (**a**,**b**) SEGA/PEDOT binary composite and (**c**,**d**) SEGA/PEDOT/MnO_2_ ternary composite; (**e**–**g**) Elemental mapping results (C, S and Mn) of the selected area in (**c**).

After the electrochemical deposition of PEDOT, the microstructure of the SEGA/PEDOT binary composite was observed. As shown in Figure 4a, the highly porous, honeycomb-like, and oriented cellular structure of SEGA is well inherited without any collapse after the deposition of PEDOT. As displayed in the SEM images at high magnification (Figure 4b), it can be found that PEDOT do not change the surface morphology of graphene cell walls significantly. And the thickness of the graphene cell walls is just slightly increased after the deposition of PEDOT, which is attributed to the relatively low mass content of PEDOT (18.8 wt %) in the composite. After electrochemical deposition of MnO_2_, the honeycomb-like and oriented cellular architecture of the SEGA is still preserved well (Figure 4c). It can be found that abundant pompon-like MnO_2_ spheres attached on the cell walls of the ternary composite (Figure 4d), which is significantly different from the smooth surface of cell walls in SEGA (Figure 3d) and SEGA/PEDOT composite (Figure 4b). In the SEM images at high magnification (Figure S2), we can see that the pompon-like spheres with diameter ~0.5 μm are composed of a quantity of MnO_2_ nanoflakes. These spheres are homogeneously distributed on the graphene cell walls even at the inner portion of the composite, which is attributed to that macroporous structure and large cell dimension of the composite enable the fast flux and uniform penetration of precursor solution into the interior zone. EDS mapping results of C, S and Mn (Figure 4e–g) also confirm the homogenous distribution of PEDOT and MnO_2_ on the graphene cell walls.

**Figure 5:**
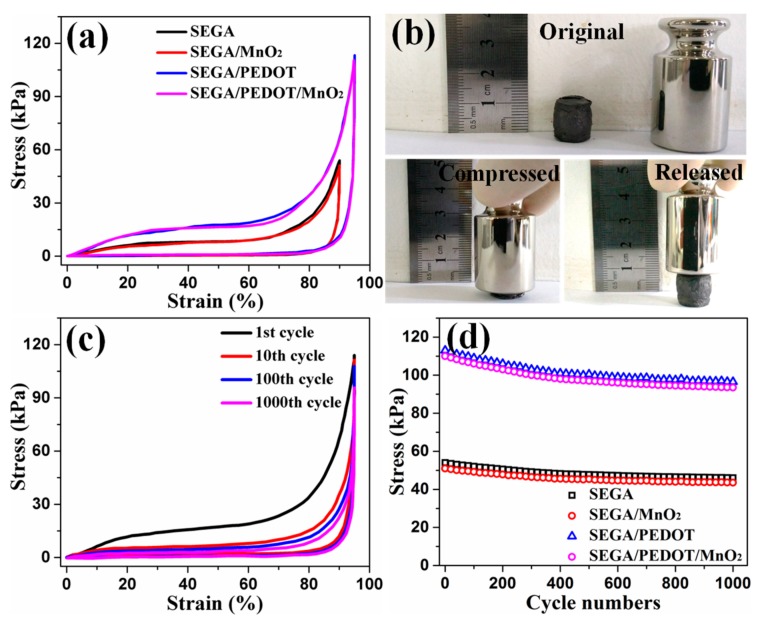
(**a**) Compressive stress-strain curves of SEGA, SEGA/MnO_2_, SEGA/PEDOT, and SEGA/PEDOT/MnO_2_ at their maximum compressive strains, respectively; (**b**) Real-time photos of the compression-recovery process of SEGA/PEDOT/MnO_2_ composite; (**c**) Compressible stress-strain curves of the 1st, 10th, 100th and 1000th cycles of SEGA/PEDOT/MnO_2_ composite at a set compressive strain of 95%; (**d**) Compressive stress values of the samples at their maximum compressive strains for 1000 cycles.

The mechanical performances of samples were tested. The stress/strain curves of SEGA, SEGA/MnO_2_ composite, SEGA/PEDOT composite and SEGA/PEDOT/MnO_2_ composite are shown in Figure 5a. As reported in the previous literatures, graphene aerogel with honeycomb-like and oriented cellular structure can present ultra-compressible ability [9,16]. It can be found that, SEGA can bear the maximum compressive strain of 90% (stress = 54 kPa) without any plastic deformation (Figure 5a). After the deposition of PEDOT, the unloading curves of SEGA/PEDOT composite at high compressive strain also can return to origin without producing residual strain (Figure 5a). It is worth noting that the maximum recoverable strain of SEGA/PEDOT composite reaches 95% (stress = 113 kPa), which is higher than that of SEGA. And the compressive stress of SEGA/PEDOT composite is higher than that of SEGA at the same strains (Figure 5a). This phenomenon is attributed to the physical reinforcement of the graphene cell walls by the uniform coating of PEDOT. The PEDOT coating layers tightly adhere on the graphene cell walls due to the strong π-π interaction between PEDOT and graphene sheets. Upon loading, the load is effectively transferred between the graphene skeleton and the PEDOT coating layers. This unique structure can help to relax the local stress and dissipate the micro-crack energy. Similar mechanisms of 3D graphene reinforced by polymer have also been mentioned in previous literatures [35,36,37].

After the deposition of MnO_2_, SEGA/PEDOT/MnO_2_ composite shows the similar stress/strain curve with SEGA/PEDOT composite (Figure 5a). And SEGA/MnO_2_ also shows the similar stress/strain curves with SEGA (Figure 5a). This indicates that the deposition of MnO_2_ does not significantly influence the mechanical performances of the backbones. As shown in the real-time photos of the compression-recovery process of SEGA/PEDOT/MnO_2_ composite (Figure 5b), the ternary composite can be squeezed into a pellet under manual compression and recover the volume without structural fatigue. This compression-tolerant ability of the SEGA/PEDOT/MnO_2_ composite is also reflected by the inner microstructure of the composite. As can be seen from Figure S3a, the initial ordered cellar structure is conformably densified while keeping the continuous configuration under compression. Once released, the SEGA/PEDOT/MnO_2_ composite rapidly recovers to the initial state without any collapse of the ordered cellar structure (Figure S3b). And MnO_2_ spheres are still tightly attached on the cell wall surface of SEGA/PEDOT without obvious peel-off after the compression-release process (Figure 3c,d). The cycle stability of the compressible ability of SEGA/PEDOT/MnO_2_ composite was also studied. As shown in Figure 5c, the first compression cycle is different from the subsequent ones showing the higher stress at the same strains. The hysteresis loop for the 10th cycle shrinks significantly compared to the first one. Since the 10th cycle, the shrink of the stress/strain curves becomes unremarkable. The decrease of the loading curves is attributed to the irreversible damage occurred in partial region of the porous structure. During the first compress-release process, the pressure leads to the bending of cell structures followed by the breakage of a few interconnected cells. For the subsequent cycles, the loading curves are gradually overlapped with each other, indicating that less and less breakdown occurred in these cycles. This phenomenon is also reported in the previous literatures [1,9,16,38]. Although there is a little failure happened during the compress-release process, the total strain was lost for only about 5% and tended to be stable after the 10th cycles (Figure S4). In addition, the maximum compressive stress of SEGA/PEDOT/MnO_2_ composite only slightly decreases to 84% of the original value (stress = 110 kPa) after 1000 compression-release cycles (Figure 5d). Therefore, the SEGA/PEDOT/MnO_2_ composite is ready for the compressible electrode materials of compression-tolerant ECs.

**Figure 6:**
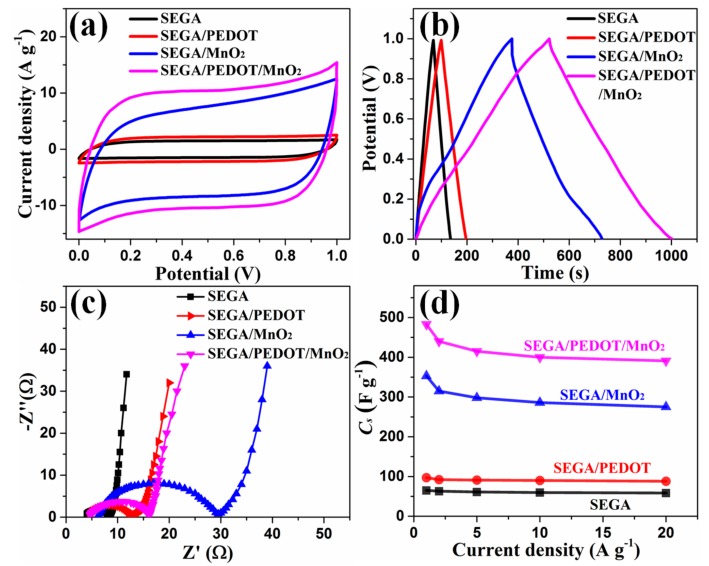
(**a**) CV curves; (**b**) GCD curves; (**c**) Nyquist impedance plots; (**d**) Rate capacity of SEGA, SEGA/PEDOT, SEGA/MnO_2_, and SEGA/PEDOT/MnO_2_, respectively.

The electrochemical performances of SEGA/PEDOT/MnO_2_ ternary composite were firstly investigated in the three-electrode system by CV and GCD tests. Figure 6a compares the CV curves of SEGA, SEGA/PEDOT, SEGA/MnO_2_, and SEGA/PEDOT/MnO_2_ electrodes at 20 mV s^−1^. The CV responses of SEGA/MnO_2_ and SEGA/PEDOT/MnO_2_ are much larger than that of SEGA and SEGA/PEDOT, indicating that MnO_2_ is mainly responsible for the high current density and enlarged area. Compared with SEGA/MnO_2_, CV curve of SEGA/PEDOT/MnO_2_ is relatively rectangular in shape and exhibits a mirror-like replication below and above the zero line, indicating the higher electrochemical performance of SEGA/PEDOT/MnO_2_. GCD curves of the different electrodes at 1 A g^−1^ are shown in Figure 6b. Compared with SEGA and SEGA/PEDOT electrodes, SEGA/MnO_2_ and SEGA/PEDOT/MnO_2_ electrodes show obvious voltage drops (*iR* drops) at beginning of discharge, which is attributed to the high resistance of MnO_2_ phase. And the discharge curve of SEGA/PEDOT/MnO_2_ electrode has less *iR* drop (0.03 V) than SEGA/MnO_2_ electrode (0.08 V), indicating that PEDOT reduces the interface resistance between graphene and MnO_2_. In agreement with the CV results, the GCD curve of SEGA/PEDOT/MnO_2_ composite holds the longest discharge time, with a specific capacitance increased from 65 F g^−1^ for SEGA to 97 F g^−1^ for SEGA/PEDOT, 353 F g^−1^ for SEGA/MnO_2_, and 483 F g^−1^ for SEGA/PEDOT/MnO_2_, respectively. This improvement of the specific capacitance for the ternary composite is attributed to the synergistic effect of the three components. In the SEGA/PEDOT/MnO_2_ composite, the SEGA provides continuously conductive network; MnO_2_ significantly improves the specific capacitance; and the middle PEDOT layer reduces the interface resistance between graphene and MnO_2_. EIS test was performed to further characterize the SEGA/PEDOT/MnO_2_ composite electrode. As shown in Figure 6c, the Nyquist plots of different electrodes are all composed of a typical semicircle in the high frequency region and a straight line in the low frequency region. The diameter of the semicircle corresponds to the charge-transfer resistance of the electrodes. It can be seen that, SEGA/PEDOT/MnO_2_ shows a significant decrease of charge-transfer resistance than that of SEGA/MnO_2_, which verifies that the middle PEDOT coating layer improves the electron transport from MnO_2_ to graphene. The rate capacity and cycle stability of the ternary composite electrode were also studied. As the current density increases from 1 to 20 A g^−1^, the specific capacitance of SEGA/PEDOT/MnO_2_ composite electrode has 81.6% retention of its initial value (Figure 6d), indicating a good rate capability. And its specific capacitance preserves 91.1% after 1000 GCD cycles at current density of 1 A g^−1^ (Figure S5), showing an excellent cycling stability.

**Figure 7:**
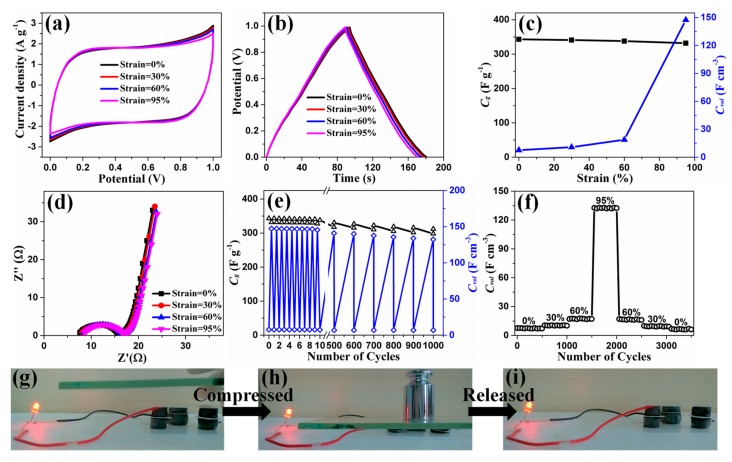
(**a**) CV curves; (**b**) GCD curves; (**c**) capacitive properties; (**d**) Nyquist impedance plots of the ECs based on SEGA/PEDOT/MnO_2_ electrodes at various compressive strains, scan rate: 20 mV s^−1^, current density: 1 A g^−1^; (**e**) The variation of gravimetric capacitances and volumetric capacitances of the ECs based on SEGA/PEDOT/MnO_2_ electrodes at original state then under compressive strain of 95% for each cycle; (**f**) Cycle performance test for 3500 charge/discharge cycles under constant compressive strains of 0%, 30%, 60%, and 95%; (**g**–**i**) Photographs of a red-light-emitting diode powered by the integrated EC unit during the compression/release process.

In order to demonstrate the electrochemical performances of SEGA/PEDOT/MnO_2_ composite under various compressive strains, we assembled the compressible all-solid-state ECs based on SEGA/PEDOT/MnO_2_ electrodes. In comparison to the liquid-electrolyte-based ECs that may suffer from the possible leakage of electrolytes, the all-solid-state ECs show enhanced safety under high-level compressive strain. As shown in Figure 7a, the CV curves of the ECs based on SEGA/PEDOT/MnO_2_ electrodes under compression state (strain = 30%, 60%, 95%) show similar characteristics with that of the ECs at the original state (strain = 0%), indicating the good electrochemical stability of the SEGA/PEDOT/MnO_2_ electrodes under compression. The GCD curves of the ECs based on SEGA/PEDOT/MnO_2_ electrodes subjected to various compressive strains show only a little slight deviation (Figure 7b), which verifies the compression-tolerant ability of the SEGA/PEDOT/MnO_2_ electrodes. This excellent performance of the electrodes arises from the synergistic effect of the three components: SEGA provides the conductive and superelastic backbone, PEDOT reduces the internal resistance and further improves the compressible ability, MnO_2_ tightly coated on the cell walls contributes the high pseudocapacitance. The roughness mechanical performances and stable microstructure of the SEGA/PEDOT/MnO_2_ composite play an important role on this compression-tolerant ability. As mentioned above, there is no significant collapse of the continuous structure (Figure S3a,b) and no obvious peel-off of MnO_2_ spheres from the cell walls of composite (Figure S3c,d) even under high-level compression, which are important for electron transport, stable conductivity and minimizing capacitance loss. Thus, ultra-compressible ability and structural robustness of the SEGA/PEDOT/MnO_2_ composite lead to high stability of pseudo reactions and charge transfer in the electrodes at high-level compressive strains.

As shown in Figure 7c, the ECs based on SEGA/PEDOT/MnO_2_ electrodes show the gravimetric capacitance of 343 F g^−1^ at original state, and retain 97% of this value at 95% compressive strain (332 F g^−1^). The gravimetric capacitance values of SEGA/PEDOT/MnO_2_ electrodes with/without compression are comparable to that of other graphene-based compressible electrode materials (Table 1). It worth noting that the volumetric capacitance of SEGA/PEDOT/MnO_2_ electrodes is dramatically improved after 60% strain, and finally reach maximum value of 147.4 F cm^−3^ under 95% strain (Figure 7c), which is much higher than other graphene-based compressible electrodes (Table 1). The remarkable improvement of volumetric capacitance results from almost unchanged gravimetric capacitance and significant increased density of the SEGA/PEDOT/MnO_2_ electrodes under high compression. When the electrodes undergo 95% compressive strain, the density of the electrodes is 20 times the original value, and the gravimetric capacitance declines by only 3%. According to the Equation (3), the volumetric capacitance of SEGA/PEDOT/MnO_2_ electrodes at compressive strain of 95% is 19.4 times that of them at uncompressed state.

The EIS of the ECs based on SEGA/PEDOT/MnO_2_ electrodes was also characterized (Figure 7d). The Nyquist plots consist of a typical semicircle in the high frequency region and a straight line at low frequency. The ECs show similar Nyquist plots in original and compressed states (strains of 30%, 60% and 95%), verifying the compression-tolerant ability. In order to study the reversible compressibility and durability of the compressible ECs with SEGA/PEDOT/MnO_2_ electrodes, cycle stability was demonstrated by GCD at 1 A g^−1^. Under both static (constant compressive strain) condition and dynamic (repeated compression/release) condition, there is only slight fluctuation of capacitances (Figure 7e). For long-term durability of ECs, the compressive strains of 0%, 30%, 60%, and 95% are each varied at 500 charge/discharge cycles and finally, recovered to a fully relaxed state (Figure 7f). The original volumetric capacitance of ECs is preserved by 80% after 3500 charge/discharge cycles with various compressive strains. In addition, for realizing the practical function of compression-tolerant ability, three compressible all-solid-state ECs were integrated into one unit and interconnected together in series. After charging for 10 min, the resultant integrated device can light up a red-light-emitting diode and works well during the compression/release process (Figure 7g–i).

## 4. Conclusions

For acquiring the compressible electrodes with both ultra-compressible ability and high specific capacitance, PEDOT and MnO_2_ were successively deposited into SEGA. Compression tests show that the maximum compressive strain of SEGA/PEDOT/MnO_2_ composite reaches 95%. The gravimetric capacitance of the SEGA/PEDOT/MnO_2_ electrodes without compression is 343 F g^−1^, and can retain 97% under 95% compressive strain. Resulting from the invariant of gravimetric capacitance of SEGA/PEDOT/MnO_2_ electrodes under compression, the volumetric capacitance of the ternary composite electrodes reaches 147.4 F cm^−3^ at 95% strain. This ternary composite paves a new way for advanced applications of ultra-compressible electrodes in the area of compression-tolerant ECs.

## Figures and Tables

**Table 1:** Comparison of graphene-based compressible electrode materials.

Materials	Uncompressed Capacitance	Maximum Compressive Strain	Compressed Capacitance	Mass content/Mass Loading of Pseudomaterials	Crystalline Phases of MnO_2_	Test Condition	Reffrence
Cross-linked graphene aerogel	90 F g^−1^	90%	130 F g^−1^	-	-	10 mV s^−1^	[2]
	0.94 F cm^−3^		13.6 F cm^−3^				
Graphene-carbon nanotube aerogel	37 F g^−1^	90%	45 F g^−1^	-	-	1 A g^−1^	[17]
	0.5 F cm^−3^		6 F cm^−3^				
Melamine foam/graphene/polypyrrole sponge	411 F g^−1^	75%	329 F g^−1^	3.92 wt %	-	10 mV s^−1^	[18]
				N.A.			
Graphene/polypyrrole foam	350 F g^−1^	50%	350 F g^−1^	N.A.	-	1.5 A g^−1^	[19]
	14 F cm^−3^		28 F cm^−3^	6.7–7.4 mg cm^−2^			
Graphene-carbon nanotube/MnO_2_ aerogel	98 F g^−1^	50%	106 F g^−1^	17 wt %	Manganite and Ramsdelite	2 mV s^−1^	[20]
	1.5 F cm^−3^		3.3 F cm^−3^	0.4 mg cm^−2^			
SEGA/PEDOT/MnO_2_ composite	343 F g^−1^	95%	332 F g^−1^	77.4 wt %	Birnessite-type	1 A g^−1^	This work
	7.6 F cm^−3^		147.4 F cm^−3^	8.6 mg cm^−^^2^			

## Supplementary Materials

The following are available online at www.mdpi.com/1996-1944/10/12/1353/s1. Table S1. Mass content and mass loading of the materials in different composites. Figure S1. XPS spectrum of Mn 2p of SEGA/PEDOT/MnO_2_ composite, Figure S2. SEM images of SEGA/PEDOT/MnO_2_ composite at high magnification, Figure S3. SEM images of SEGA/PEDOT/MnO_2_ composite corresponding to the loading status and unloading status, Figure S4. A part of the stress-strain curves (strain ≤ 10%) of SEGA/PEDOT/MnO_2_ composite during the measurement of the cycle stability at a set strain of 95%, Figure S5. Cycle stability of SEGA/PEDOT/MnO_2_ composite in the three-electrode system.
