# Supplementary material for: Superelastic Graphene Aerogel/Poly(3,4-Ethylenedioxythiophene)/MnO2 Composite as Compression-Tolerant Electrode for Electrochemical Capacitors
Source: Materials (Basel). 2017 Nov 24;10(12):1353. doi: 10.3390/ma10121353 (PMC5744288; doi:10.3390/ma10121353)
Supplement: Supplementary file 1 [file materials-10-01353-s001.pdf]

# Supplementary Materials: Superelastic Graphene Aerogel/Poly(3,4-Ethylenedioxythiophene)/$MnO_2$ Composite as Compression-Tolerant Electrode for Electrochemical Capacitors

**Peng Lv *, Yaru Wang, Chenglong Ji, Jiajiao Yuan**

**Table S1.** Mass content and mass loading of the pseudomaterials in different composites.

| Composites | PEDOT | | $MnO_2$ | |
|---|---|---|---|---|
| | Mass Content | Mass Loading | Mass Content | Mass Loading |
| SEGA/PEDOT | 44.9 wt % | 2.1 mg $cm^{-2}$ | - | - |
| SEGA/$MnO_2$ | - | - | 71.0 wt % | 6.3 mg $cm^{-2}$ |
| SEGA/PEDOT/$MnO_2$ | 18.8 wt % | 2.1 mg $cm^{-2}$ | 58.6 wt % | 6.5 mg $cm^{-2}$ |

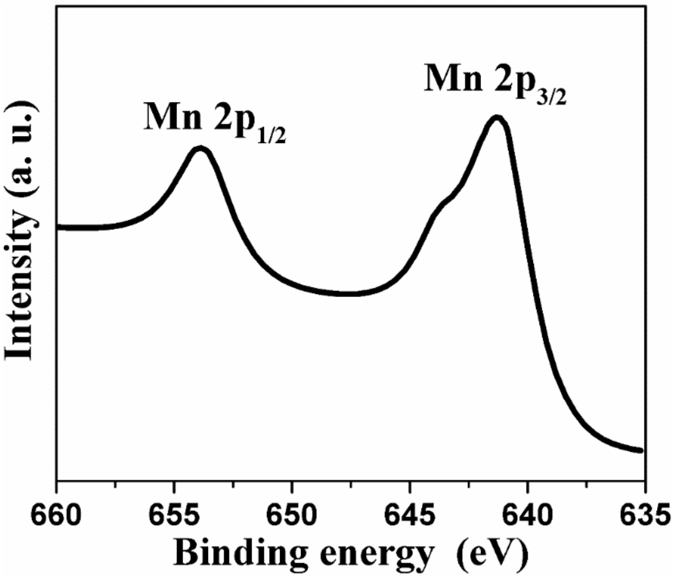


**Figure S1.** XPS spectrum of Mn 2p of SEGA/PEDOT/$MnO_2$ composite.

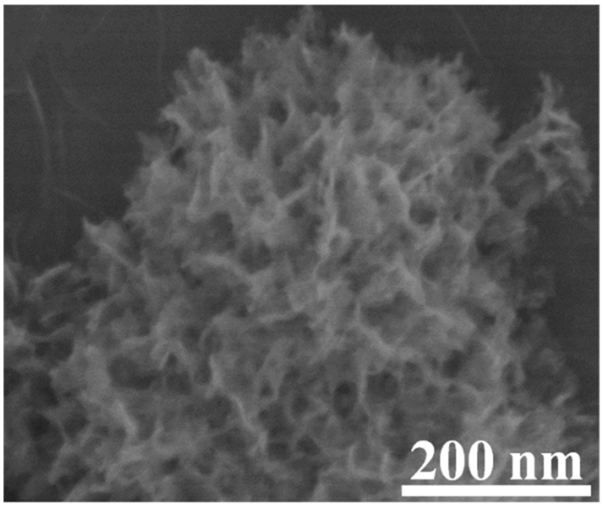


**Figure S2.** SEM images of SEGA/PEDOT/$MnO_2$ composite at high magnification.

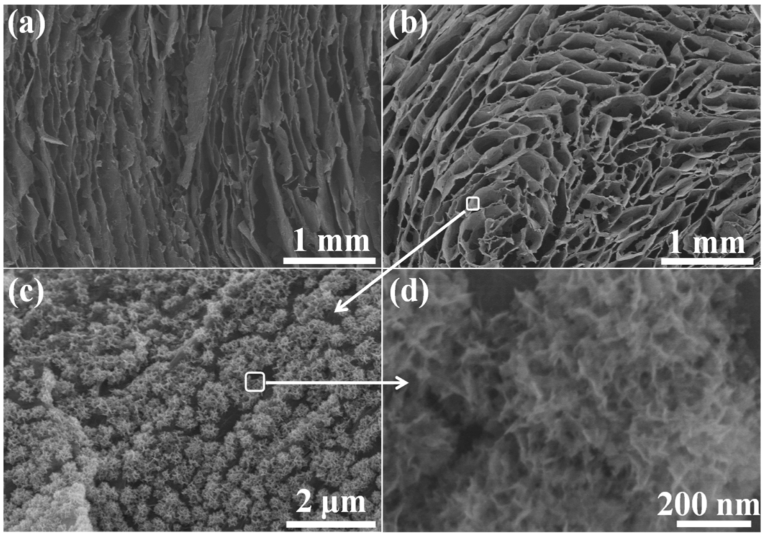


**Figure S3.** SEM images of SEGA/PEDOT/$MnO_2$ composite corresponding to the (**a**) loading status and (**b**–**d**) unloading status.

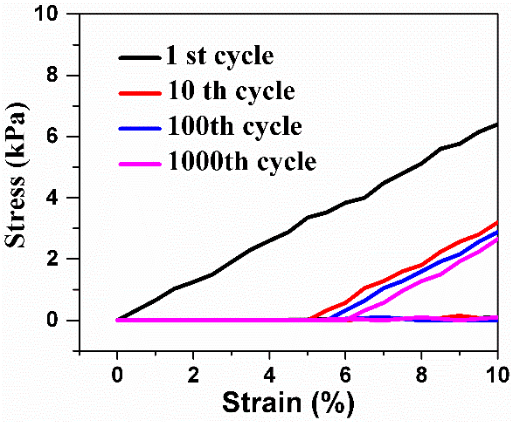


**Figure S4.** A part of the stress-strain curves (strain≤10%) of SEGA/PEDOT/$MnO_2$ composite during the measurement of the cycle stability at a set strain of 95%.

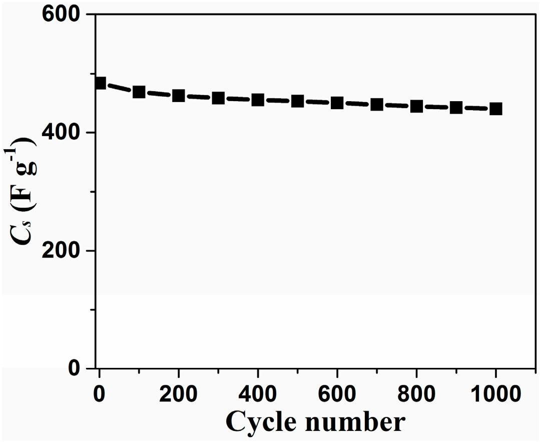


**Figure S5.** Cycle stability of SEGA/PEDOT/$MnO_2$ composite in the three-electrode system.

© 2017 by the authors. Submitted for possible open access publication under the terms and conditions of the Creative Commons Attribution (CC BY) license (http://creativecommons.org/licenses/by/4.0/).
